# Ceftriaxone-Induced Thrombocytopenia: A Case Report on a Rare Medical Condition

**DOI:** 10.7759/cureus.83811

**Published:** 2025-05-09

**Authors:** Badri Aryal, Audrey Simmons, Giri Ekkurthi, Bibechana Panthi, Oscar Ruben Pena-Vizcarra

**Affiliations:** 1 Internal Medicine, John H. Stroger, Jr. Hospital of Cook County, Chicago, USA; 2 Internal Medicine, Chicago Medical School, Rosalind Franklin University, Chicago, USA; 3 Internal Medicine, Lumbini Medical College and Teaching Hospital, Palpa, NPL

**Keywords:** adverse effect, ceftriaxone adverse effects, clinical case report, drug-induced thrombocytopenia, heparin-induced thrombocytopenia (hit), sepsis

## Abstract

We present a case of antibody-confirmed drug-induced thrombocytopenia (DITP) caused by ceftriaxone. DITP is a rare but potentially life-threatening cause of isolated thrombocytopenia in the inpatient setting, often leading to critically low platelet levels. The underlying mechanism is believed to be an immune-mediated response, where drug-dependent antibodies bind to glycoproteins on the platelet membrane in the presence of the offending drug, resulting in platelet destruction and thrombocytopenia. We describe a case of ceftriaxone-induced thrombocytopenia in a patient being treated for aspiration pneumonia complicated by sepsis. To the best of our knowledge, only eight similar cases have been reported in the literature.

## Introduction

Thrombocytopenia is defined as a platelet count of less than 150,000 platelets/µL of blood [1]. The reference range is 150,000-450,000/μL. A significant reduction in the number of platelets (less than 50,000/μL) may lead to the occurrence of petechiae. Spontaneous bleeding can occur at a platelet count of less than 10,000/μL [1]. The causes of isolated thrombocytopenia are diverse, and identifying the underlying cause is critical to prevent potentially life-threatening complications. Clinical symptoms can vary widely, ranging from mild manifestations such as petechiae and nosebleeds to more serious complications, including gastrointestinal and intracranial bleeding.

DITP is an idiosyncratic immune-mediated reaction that occurs due to the production of antibodies against platelet glycoproteins, which are formed in the presence of sensitizing drugs, leading to platelet destruction [2,3]. This differs from the hapten-mediated immune mechanism seen in heparin-induced thrombocytopenia (HIT), where antibodies are directed against complexes formed between heparin and platelet-factor 4 surface glycoprotein, leading to platelet activation and thrombus formation. Common drugs associated with DITP include abciximab, carbamazepine, eptifibatide, heparin, ibuprofen, mirtazapine, oxaliplatin, penicillin, quinine, quinidine, rifampicin, suramin, tirofiban, trimethoprim-sulfamethoxazole, and vancomycin [4]. Diagnosing DITP requires exclusion of all other alternative causes (infections, sepsis, vitamin/mineral deficiencies, HIT, etc.) as well as the correlation between clinical presentation, laboratory findings, and timing of drug initiation [3]. DITP can also be confirmed through the presence of drug-dependent platelet antibodies. However, not all suspected drug-induced thrombocytopenia (DITP) cases have reported positive serology. Because such testing is not always available and requires significant time, it is not possible to wait for test results before deciding whether to discontinue a potential causative drug. Given this, if there is high clinical suspicion for DITP, the offending medication should be discontinued before serology confirmation or even in the setting of negative serology to prevent life-threatening levels of thrombocytopenia.

Ceftriaxone-induced thrombocytopenia is a rare cause of DITP. The Oklahoma University database reported only six cases from 1991 to 2018 [5]. Two other case reports are published in the literature [6,7].

## Case presentation

We present a case of a 61-year-old man with a medical history of hypertension, heart failure with reduced ejection fraction, atrial fibrillation on apixaban, and alcohol use disorder, who presented to the emergency department (ED) of our hospital with worsening abdominal pain for one day. His pain was acute in onset, localized to the upper part of the abdomen, with some radiation to the back, and was associated with nausea and multiple episodes of nonbilious and nonbloody vomiting. He was not having any hematemesis or melena, diarrhea, or constipation. He denied fever or chills. He had no chest pain, shortness of breath, cough, or sputum production. He did not endorse orthopnea, paroxysmal dyspnea, palpitations, or lower extremity swelling. He endorsed drinking five to six drinks of alcohol per day for the last few years; however, he denied smoking or illicit drug use. His last drink was one day before the presentation. He had no prior history of thrombocytopenia or bleeding disorder.

On initial examination, vital signs were notable for a blood pressure of 105/60 mmHg, pulse rate of 59 per minute, respiratory rate of 18 per minute, and temperature of 37°C. Initial physical examination was notable for mild tenderness in the upper abdomen but was otherwise unremarkable. Laboratory work-up showed sodium of 141 mEq/L, potassium of 2.4 mEq/L, blood urea nitrogen of 11 mg/dL, and creatinine of 0.7 mg/dL. Complete blood count was notable for hemoglobin of 13 g/dL with hematocrit of 45%, white blood cell count of 11k/μL, and platelets of 240k/μL. Lipase was elevated to 2,174 U/L. Table 1 summarizes the initial lab workup done in the emergency room. Computed tomography of the abdomen and pelvis showed findings suggestive of acute pancreatitis. The patient was started on IV fluids. While awaiting admission in the ED, he also developed severe withdrawal symptoms, and he was subsequently admitted to the medical intensive care unit (MICU) for the management of severe alcohol withdrawal in addition to acute pancreatitis.

**Table 1:** Labs on initial presentation to the emergency room Laboratory value on admission shows normal platelet levels of 240k/μL

Labs	Values
Sodium	141 mEq/L
Potassium	2.4 mEq/L
Blood urea nitrogen	11 mg/dL
Creatinine	0.7 mg/dL
White blood cells count	11k/μL
Hemoglobin	13 g/dL
Hematocrit	45%
Platelets count	240k/μL
Total protein	7.1 g/dL
Albumin	3.9 g/dL
Total bilirubin	1.2 mg/dL
Direct bilirubin	0.2 mg/dL
Alkaline phosphatase	141 U/L
Gamma glutamyl transferase	808 U/L
Aspartate transaminase	88 U/L
Alanine transaminase	27 U/L
Lactate dehydrogenase	165 U/L
Lipase	2,174 U/L
Troponins	<0.030 ng/mL
Brain natriuretic peptide	120 pg/mL

**Figure 1:**
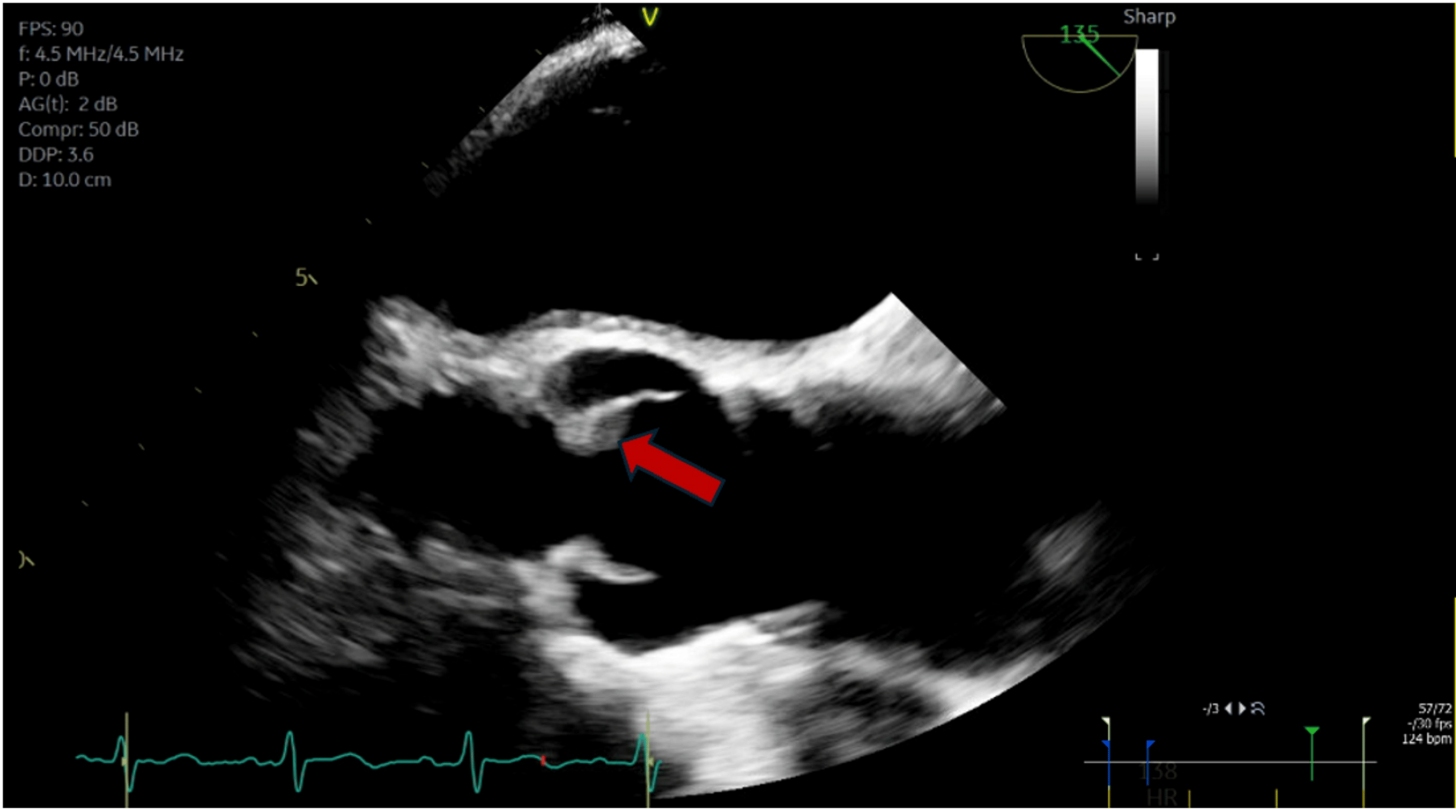
TEE showing a 6-mm fixed lesion (red arrow) on the ventricular aspect of the noncoronary cusp of the aortic valve The presence of this echocardiographic finding in the setting of bacteremia raised the suspicion of infective endocarditis, for which the patient was continued on a prolonged course of ceftriaxone. The echocardiographic finding persisted despite the antibiotic therapy and patient clinical improvement. It was labeled as a valve thickening on a follow-up echocardiography read TEE: transesophageal echocardiography

The patient was transferred to the general medicine floor after the improvement of his withdrawal symptoms. The hospital course was complicated by aspiration pneumonia and sepsis, for which he was readmitted to MICU. He was started on broad-spectrum antibiotics, which were later switched to ceftriaxone after the blood culture grew *Streptococcus sanguinis* sensitive to ceftriaxone. Transthoracic echocardiography (TTE) was unremarkable. However, transesophageal echocardiography showed a 6-mm fixed lesion on the ventricular aspect of the noncoronary cusp of the aortic valve (red arrow in Figure 1). The infectious disease (ID) team was consulted. Because of the bacteremia and echocardiography findings concerning endocarditis, ID recommended prolonged antibiotic treatment with ceftriaxone. He was also started on a heparin drip for continued anticoagulation for his atrial fibrillation.

**Figure 2:**
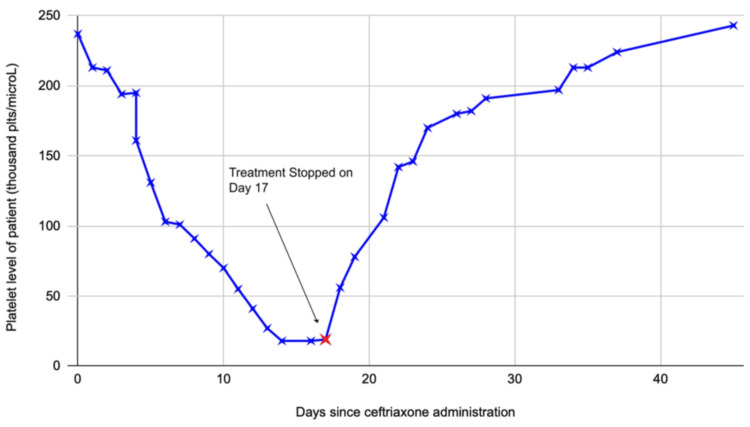
Platelet count trends in relation to when ceftriaxone therapy was initiated. Arrowhead points to the timing of stopping ceftriaxone As depicted in the graph, a progressive rise in platelet levels was observed following cessation of ceftriaxone, with platelet levels returning to normal in 7-10 days

The patient had a platelet count of 240k/μL at the initial presentation. One week after the MICU admission, the patient had a progressive decline in platelet levels. The platelet counts dropped daily by 10,000-20,000 until reaching a nadir of 18k/μL (as shown in the graph in Figure 2). HIT was initially suspected, given the 4T score of 4, suggesting intermediate probability, for which the hematology team was consulted. Heparin was immediately stopped and switched to argatroban. However, platelet levels did not improve even after stopping heparin products. After a few days, HIT antibodies and serotonin release assay (SRA) came back negative. Sepsis-induced thrombocytopenia was then considered as a differential diagnosis. The patient improved clinically over the next one week, and the bacteremia was cleared on repeated blood cultures. The patient, however, continued to remain thrombocytopenic with no improvement in platelet counts even after the resolution of sepsis. The patient was transferred to the general medicine floor and continued on ceftriaxone.

The possibility of DITP was considered as the underlying etiology of thrombocytopenia after ruling out other possible causes. A thorough review of medication administration records showed that the platelet levels started dropping after five days of ceftriaxone initiation (as demonstrated in the graph in Figure 2), because of which ceftriaxone-dependent platelet immunoglobulin G (IgG) antibodies were sent, which later came back positive. The ID team was reconsulted for alternative antibiotics, who recommended obtaining a repeat TTE. Follow-up TTE showed persistence of the aortic valve lesion, which was deemed to be a valve thickening rather than vegetation by the cardiology consult team at this time. After an interdisciplinary discussion, a decision was made to discontinue ceftriaxone as the concerns for endocarditis were very low at this point.

After ceftriaxone was stopped (day 17 on Figure 2), a gradual uptrend in platelet levels was seen. Based on the exclusion of other potential differential diagnoses, temporal association with ceftriaxone use, positive ceftriaxone-dependent IgG antibodies, and improvement of platelet levels with discontinuation of the drug, we concluded that our patient had ceftriaxone-induced thrombocytopenia. The patient had no bleeding manifestations throughout hospitalization, and his hemoglobin remained stable. Hence, he did not require a platelet transfusion.

## Discussion

DITP is a rare cause of isolated thrombocytopenia in the inpatient setting, often leading to critically low platelet levels, which can be life-threatening. DITP caused by ceftriaxone is not usually thought of as a potential cause of thrombocytopenia in hospitalized patients, often leading to delayed diagnosis and intervention [4,7]. Recommended criteria for evaluating DITP are based on the following factors: temporal association, exclusion of alternative causes, specific laboratory testing, improvement/resolution upon stopping the drug, and drug rechallenge if deemed safe [3]. Our patient met all criteria except drug rechallenge, which was not tested in light of possible life-threatening thrombocytopenia. We did not find studies specific to ceftriaxone in the literature. However, DITP is believed to be an immune-mediated reaction due to the production of antibodies against platelet glycoproteins, which are formed in the presence of sensitizing drugs, leading to platelet destruction [2,3].

The first step in evaluating inpatient thrombocytopenia is to rule out pseudothrombocytopenia, which can be done by visualization of platelet clumping on peripheral blood smears. Our patient had true thrombocytopenia. HIT should be considered if there is a decline in platelet count following the initiation of the heparin product. It can be done based on the 4 T's pretest probability score for HIT, which is based on four criteria: thrombocytopenia degree, timing of onset of thrombocytopenia, thrombosis or other sequelae, and other possible causes [8]. Our patient had a 4 T score of 4, indicating intermediate clinical probability of HIT. However, thrombocytopenia persisted, and platelet levels continued to decline even after the cessation of heparin. Additionally, HIT antibodies and SRA were negative.

Sepsis is also a common cause of thrombocytopenia in hospitalized patients. Thrombocytopenia due to sepsis improves over 5-10 days [9] and usually resolves with treatment of sepsis [10]. Our patient improved clinically over the course of hospitalization, and the bacteremia was cleared on repeated blood cultures. He continued to remain thrombocytopenic with no improvement in platelet counts even two weeks after the resolution of sepsis. Other infectious causes of thrombocytopenia include HIV, hepatitis B and C, Epstein-Barr virus, and cytomegalovirus, all of which were negative in our patient. Thrombocytopenia can sometimes be caused by nutritional deficiencies such as vitamin B12, folate, and copper, which were normal in our patient.

Thrombocytopenia can be caused by alcohol use. It is usually transient and returns to normal levels within one week of cessation [11]. Our patient also had a history of chronic alcohol use. However, his platelet count was normal at the time of hospitalization and started to drop around six days after the hospitalization. Chronic liver disease or cirrhosis is another cause of thrombocytopenia [12]. However, the patient's laboratory tests and imaging findings did not reveal chronic liver disease.

Microangiopathic causes, such as thrombotic thrombocytopenic purpura and hemolytic uremic syndrome, are other causes of thrombocytopenia. In our patient, there was no evidence of schistocytes on peripheral blood smear or findings suggestive of hemolytic anemia. Additionally, he had no signs and symptoms of autoimmune disease, and antinuclear antibody was negative. He had no thrombosis or other concerning features, so disseminated intravascular coagulation, antiphospholipid syndrome, and paroxysmal nocturnal hemoglobinuria were not considered differentials. Bone marrow biopsy was not pursued as the platelet count improved after stopping ceftriaxone.

Management of DITP involves stopping the causative drug, which usually leads to improvement of platelet counts. Patients with severe thrombocytopenia or spontaneous bleeding might require platelet transfusion. Corticosteroids are often used in unexplained thrombocytopenia because it is difficult to exclude immune thrombocytopenia [2]. When DITP is suspected, it is appropriate to abruptly stop corticosteroid therapy after the platelet count returns to normal [2]. Drug-dependent antibodies can persist for several years, and patients should be advised to avoid the drug that caused thrombocytopenia indefinitely [2]. Our patient's platelet counts improved significantly after stopping ceftriaxone, and he did not require a platelet transfusion.

This case is unique because the patient had multiple comorbid conditions, which could be falsely labeled as a potential cause of thrombocytopenia, leading to the delay in diagnosis of DITP.

## Conclusions

Acquired isolated thrombocytopenia in hospitalized patients can arise from various etiologies. We report a rare case of ceftriaxone-induced thrombocytopenia in the context of sepsis secondary to aspiration pneumonia. This case underscores the importance of maintaining a high index of suspicion for DITP, particularly when thrombocytopenia remains unexplained even after ruling out more common differential diagnoses. In such scenarios, a comprehensive review of the patient’s medication history is essential to ensure timely identification and management of potential drug-related adverse effects.
